# Role of Spastin in Apical Domain Control along the Rhabdomere Elongation in *Drosophila* Photoreceptor

**DOI:** 10.1371/journal.pone.0009480

**Published:** 2010-03-03

**Authors:** Geng Chen, Garrett P. League, Sang-Chul Nam

**Affiliations:** Department of Biology, Baylor University, Waco, Texas, United States of America; University of Texas MD Anderson Cancer Center, United States of America

## Abstract

**Background:**

Mutations in *spastin* are the most common cause of hereditary spastin paraplegia, a neurodegenerative disease. In this study, the role of *spastin* was examined in *Drosophila* photoreceptor development.

**Methodology/Principal Findings:**

The *spastin* mutation in developing pupal eyes causes a mild mislocalization of the apical membrane domain at the distal section, but the apical domain was dramatically reduced at the proximal section of the developing pupal eye. Since the rhabdomeres in developing pupal eyes grow from distal to proximal, this phenotype strongly suggests that *spastin* is required for apical domain maintenance during rhabdomere elongation. This role of *spastin* in apical domain modulation was further supported by *spastin's* gain-of-function phenotype. Spastin overexpression in photoreceptors caused the expansion of the apical membrane domain from apical to basolateral in the developing photoreceptor. Although the localizations of the apical domain and adherens junctions (AJs) were severely expanded, there were no defects in cell polarity.

**Conclusions/Significance:**

These results strongly suggest that *spastin* is essential for apical domain biogenesis during rhabdomere elongation in *Drosophila* photoreceptor morphogenesis.

## Introduction

Hereditary spastic paraplegia (HSP) comprises a heterogeneous group of neurological disorders [1]. The autosomal dominant form of hereditary spastic paraplegia (AD-HSP) is a neurodegenerative disorder. In 40% of families with the pure form of AD-HSP, the disease is linked to mutations of a gene encoding spastin [2]. It was suggested that the strong neurodegenerative defects observed in patients are caused by a primary defect of spastin in neurons [3]. Spastin is a microtubule-severing AAA ATPase [2], [4], [5] involved in constructing neuronal and non-centrosomal microtubule arrays [6], [7].

*Drosophila* is an attractive system for testing *in vivo* function of *spastin*. *Drosophila* contains a highly conserved spastin homolog [8]. Recently, functions of *Drosophila* spastin were analyzed in developing neuromuscular junctions using genetic mutational studies. Together, these data provide the first *in vivo* evidence that Spastin regulates microtubule stability and show that this function of Spastin strongly modulates both synaptic architecture and neurotransmission strength in neuromuscular junctions [9], [10]. Recently, motor axon outgrowth defects were observed using the knock-down study in zebra fish [7].

**Figure 1:**
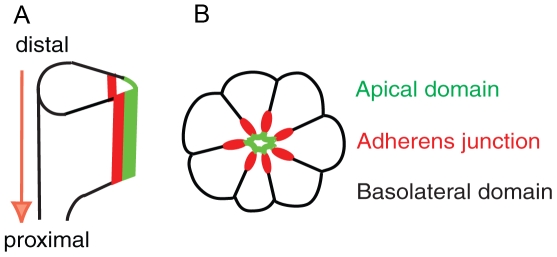
Morphogenesis of *Drosophila* pupal photoreceptors. (A) Side view of developing photoreceptors at mid-stage of pupal development. The photoreceptors elongate from distal to proximal (arrow). (B) Cross-section of mid-stage pupal photoreceptors. Apical domain (green) localizes apical to AJ (red) in the center of a photoreceptor cluster.

In this study, we analyzed the function of Spastin in photoreceptor morphogenesis of *Drosophila* eye. Photoreceptor cells are formed in eye imaginal discs of third-instar larvae. During pupal development, photoreceptor cells undergo the distal to proximal elongation (Figure 1A) [11] and the apical membrane domain localizes at the center of the photoreceptor clusters surrounded by the AJ and basolateral domains (Figure 1B) [12], [13]. Crumbs (Crb) is required for extension of photoreceptors along the distal-proximal axis of the photoreceptor cell, although it is not essential for establishing apical basal cell polarity [12], [13]. The mammalian homolog of Crb, *CRB1*, is also localized to the inner segment of photoreceptors, the structure analogous to the rhabdomere stalk, between the outer segment and the AJ [12]. Furthermore, mutations in *CRB1* causes retinal diseases including retinitis pigmentosa 12 and Leber Congenital Amaurosis in humans [14], [15]. One of the important questions is how the apical Crb domain is regulated during the rhabdomere elongation.

Here we analyzed the functional role of Spastin in the localization of apical Crb domain during photoreceptor morphogenesis. We found that *spastin* mutant photoreceptors display severely disrupted morphogenesis with dramatic reduction of apical membrane including the Crb domain. Our data suggest that *spastin* is essential for the Crb membrane domain modulations and for the morphogenesis of the developing photoreceptors.

## Results

### Localization of Stable Microtubules in *Drosophila* Pupal Photoreceptors

Previously, the microtubules in developing *Drosophila* photoreceptors were reported in the third-instar larval eye discs [16], [17], [18]. Nuclear positioning or migration functions were analyzed in the larval eye discs with the microtubule-dependent genes including *klarsicht* [16], *dynactin* [17], and *lissencephay1* [18].

Here, we examined whether the *Drosophila* pupal photoreceptor cells may have any stable microtubules. To identify stable microtubules, we used a monoclonal antibody directed against acetylated-tubulin [19], which specifically labels stabilized microtubules in cilia and axons [19], [20]. Since the acetylation of microtubules is correlated with the stabilization [21], [22], this antibody is a reliable marker to check the stable microtubules [19].

Before 45% pupal developmental (pd) stage, Crb remains at the apical surface of photoreceptors. At 45% pd (Figure 1), Crb begins to concentrate at the subapical region, which later develops into rhabdomere stalk domain [11]. Therefore, the *Drosophila* pupal eyes at 45% pd provides an ideal system to analyze the each membrane domain and cell polarity proteins' specific targeting.

**Figure 2:**
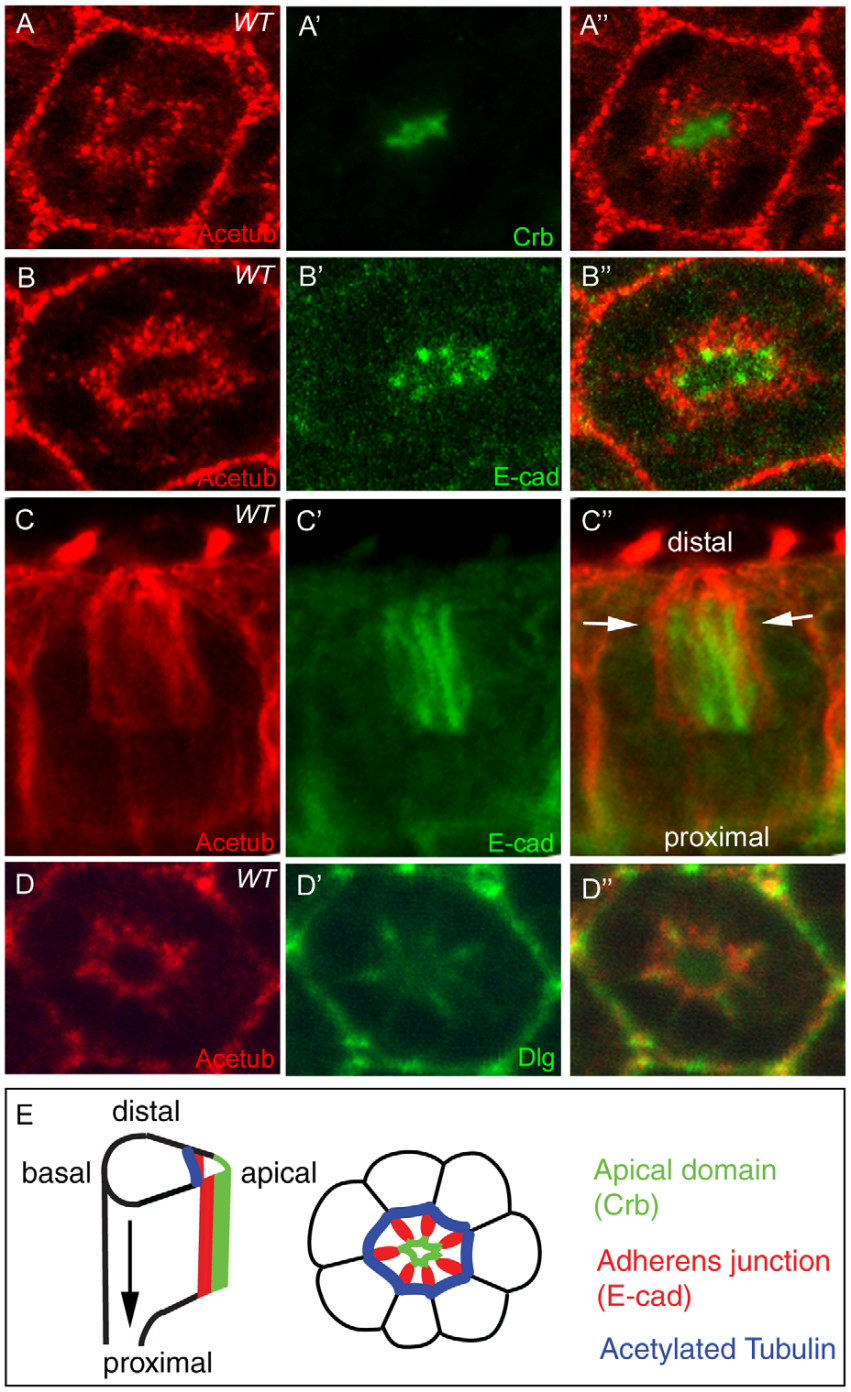
Acetylated microtubules in *Drosophila* pupal photoreceptors. Localization of acetylated microtubules in mid-stage (45% pd) of pupal eyes were examined. (A) Pupal eyes were stained with acetylated-tubulin (Acetub, red) and Crb (green). The acetylated-tubulin (Acetub, red) localizes more basal to the apical domain (Crb). (B) Acetylated microtubules (red) localizes more basal to the AJ (E-cad, green). (C) Side view of (B) shows the acetylated microtubules (red, arrows) are wrapping the AJ (E-cad, green). (D) Acetylated microtubules localize more apical to the basolateral domain (Dlg, green). (E) Schematic diagram of the localization of stabilized microtubules in pupal photoreceptors. The apical markers (Crb) localize at the apical domain (green). The E-cad localizes at AJ (red) which are more basal to the apical domain. The acetylated-tubulin (blue) localizes at the outside from the AJs (red).

The developing pupal eyes at 45% pd stage were checked by the anti acetylated-tubulin antibody. The acetylated-tubulin is highly enriched in the basal to apical domain (Figure 2A), between and more basal to the AJ (E-cad) (Figure 2B and C), and in between and more apical to the basolateral domain (Discs large, Dlg) (Figure 2D). Based on the comparative localization information using the apical, AJ, and basolateral membrane domain markers (Figure 2A–D), we propose a model in which the stable microtubules localize on the outside of AJs in developing pupal eyes (Figure 2E). Since the regular anti-α-tubulin or anti-β-tubulin antibody shows a similar pattern to the acetylated-tubulin antibody (data not shown), the microtubules in the mid-stage pupal photoreceptors exist in mostly acetylated and stabilized forms. Previously, the microtubule at the base of the rhabdomere was reported using a regular anti-α-tubulin, although its relative localization was not known [23]. But, here we identified the previously identified microtubules in pupal eyes exist as acetylated and stabilized forms. Also, we precisely identified the exact location of the stable microtubules in developing pupal eyes using the several markers of apical domain, AJ and basolateral junction. Given the presence of acetylated microtubules in the *Drosophila* photoreceptors, these stable microtubules may have some potential functions for photoreceptor development.

### Localization of Spastin in *Drosophila* Pupal Photoreceptors

**Figure 3:**
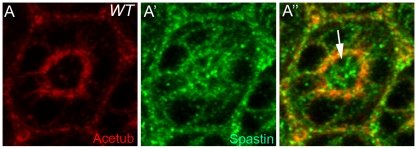
Localization of Spastin in *Drosophila* pupal photoreceptors (45% pd). (A) Spastin (A′, green) localizes not only at acetylated microtubules (A, red), but also at the apical membrane domain (A″, arrow) of the mid-stage pupal eyes.

In mammals, Spastin has been shown to modulate the microtubule cytoskeleton [24]. The *Drosophila* homolog of Spastin appears to affect the abundance and distribution of acetylated microtubules in *Drosophila* neuromuscular junctions [9]. To identify one of the potential functions of the stable microtubules in *Drosophila* photoreceptor development, the subcellular localization of Spastin in the mid-stage developing pupal eyes (45% pd) was examined by immunostaining and confocal microscopy. Spastin is highly enriched in the stable microtubules (Figure 3), as well as in the apical membrane domain (Figure 3, arrow). The specificity of Spastin staining was further confirmed by the absence of the staining in the *spastin^5.75^* [10] protein-null mutant clones (data not shown). This data strongly indicates Spastin's potential role in the stable microtubules and/or apical domain morphogenesis in *Drosophila* pupal photoreceptors.

### Spastin Is Required in Apical Domain along the *Drosophila* Photoreceptor Elongation

**Figure 4:**
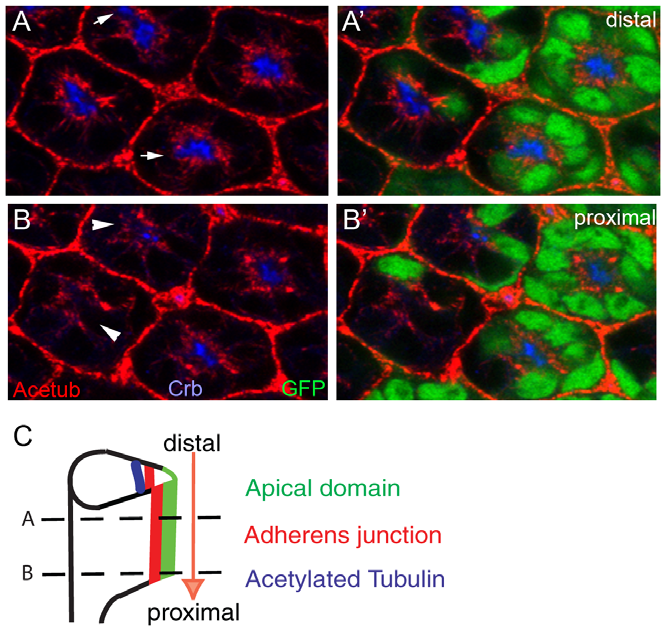
Spastin is essential for photoreceptor morphogenesis in the mid-stage developing pupal eyes. (A, B) Pupal eyes (45% pupal stage) with *spastin^5.75^* null mutant clones marked by the absence of the GFP (green). Acetylated tubulin (Acetub, red) was decreased or destabilized in the absence of the spastin. Crb (blue) is mislocalized at the distal section (A, arrows) and almost absent at the proximal section (B, arrow-heads) from the same pupal eye. (C) Developing pupal photoreceptors elongate from distal to proximal direction. Distal (A) and proximal (B) sections were marked by dashed-lines.

To examine whether Spastin is required for photoreceptor morphogenesis in mid-stage pupal eye development, we generated mosaic eyes of a null mutation of spastin, spastin^5.75^ [10], using the FLP/FRT-based genetic mosaic technique [25]. We have found that the *spastin* mutation slightly affected Crb localization (fluorescence intensity: 50±10% reduction, examined ommatidia numbers = 100, Figure 4A) at the distal section, but caused the almost complete loss of the Crb at the proximal section of the same pupal eye (0%, n>200, Figure 4B). We analyzed other apical markers of Stardust (Sdt) and Dpatj which showed the same localization defects like Crb's (data not shown). Since the rhabdomere grows from distal to proximal in developing pupal eyes (Figure 4C, arrow), this mutant phenotype of *spastin* strongly suggests that Spastin is specifically required for the apical membrane domain, including Crb, in addition to rhabdomere growth from distal to proximal during photoreceptor morphogenesis. This type of rhabdomere elongation defect was found in the cases of c*rb* and *par-1* mutations [13], [26], [27].

Similar differential defects along the distal-proximal axis were observed in the case of the stable microtubules. The stable microtubules are relatively normal at the distal section (10±10% reduction, n = 100, Figure 4A, red), but the stable microtubules are reduced at the proximal section of spastin mutants (50±10% reduction, n = 100, Figure 4B, arrowheads). It was noticed that the complete loss of Crb occurred even in the presence of the acetylated microtubules (Figure 4B, arrowheads), which strongly indicates that the loss of Crb was probably not caused by the absence of the stable microtubules. Instead, this data strongly suggests that the loss of Crb at the proximal section of spastin mutants was caused by a direct effect of the spastin mutation, rather than by an indirect influence of the stable microtubules. One possible alternative mechanism is that the spastin mutant photoreceptors fail to extend fully because of subtle defects in the microtubule cytoskeleton, which would indirectly result in the observed lack of Crb.

### Overexpression of Spastin Causes Apical Domain Expansion of *Drosophila* Pupal Photoreceptor

**Figure 5:**
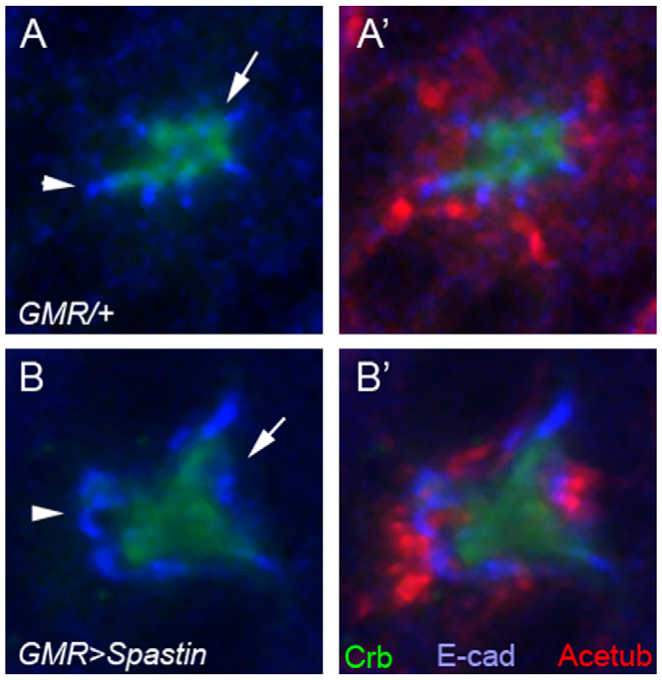
Overexpression of spastin causes the apical domain expansions. Pupal eyes (45% pd) with spastin overexpression driven by *GMR-GAL4* at 22°C were examined by Crb (green, apical domain marker), E-cad (blue, AJ marker) and Acetylated-tubulin (Acetub, red). (A) control, *GMR-GAL4/+* (B) *GMR-GAL4/UAS-spastin*. The expanded Crb domain (green, arrow) and E-cad (blue, arrowhead) were caused by the spastin overexpression (B).

The loss-of-function analysis of the *spastin* mutation (Figure 4) strongly suggests that *spastin* might affect the apical membrane domain for photoreceptor morphogenesis. Next we analyzed the gain-of-function analysis of *spastin* using eye-specific GAL4 lines, *GMR-GAL4* [28], to increase the Spastin level in the photoreceptors. We employed the established *UAS-Spastin* [10] to examine the effects of Spastin overexpression for photoreceptor morphogenesis. Spastin overexpression in the mid-stage pupal photoreceptors dramatically expanded the apical membrane domains (300±100% expansion, n = 100, Figure 5B, green), with concurrent mislocalization/expansion of the AJs (300±100% expansion, n = 100, Figure 5B, blue) from the apical center of the photoreceptor. Although the mislocalization of apical and AJs was dramatic, there were no defects in cell polarity since the Crb (apical marker) still localized more apically compared to the E-cad (AJ marker) (Figure 5B). Our results strongly suggest that Spastin specifically controls the apical membrane domain during pupal eye development based on the loss-of-function (Figure 4) and gain-of-function (Figure 5) phenotypes of *spastin*. Although the apical domains were strongly expanded based on the overexpression of spastin, the stable microtubules were not much affected (Figure 5B), indicating that apical expansion might be caused by the direct effect of spastin overexpression and not by any indirect effects caused by differences in the stable microtubule levels.

## Discussion

In animal photoreceptor cells, the surface membrane is enlarged for the storage of opsin photopigment. Insect eyes use an actin-based structure for surface membrane enlargement, but mammalian eyes use microtubule-based structure. We examined whether the *Drosophila* photoreceptor cells may have any microtubule-based structures. There is a distinctive structure which is specifically labeled by anti-acetylated-tubulin antibody in the developing photoreceptors of *Drosophila*. Given the specific localization of stable microtubules in developing pupal *Drosophila* photoreceptors, these subcellular structures might provide a functional role for photoreceptor morphogenesis.

Spastin is an ATPase that binds microtubules and localizes to the spindle pole and distal axon in mammalian cell lines. Furthermore, its *Drosophila* homolog, *Drosophila spastin*, has recently been shown to regulate microtubule stability and synaptic function at the *Drosophila* larval neuromuscular junction [9], [10]. Our genetic analysis of the spastin mutation strongly indicates that spastin not only modulates the microtubules, but also modulates the apical Crb membrane domain during rhabdomere elongation (Figure 4). The apical membrane modulation activity of spastin was further confirmed by spastin overexpression which caused a dramatic expansion of the apical membrane domain (Figure 5).

Based on the highly concentrated localization of Spastin in the apical domains of the photoreceptors (Figure 3), we propose that the apically localized Spastin might control the apical Crb domains. This apical domain-specific function of Spastin is based on the following results; (i) Apical domain localization of Spastin (Figure 3), (ii) Loss of spastin causes apical domain defects (Figure 4), and (iii) Overexpression of spastin causes apical domain expansions (Figure 5). But, we cannot exclude another possibility of the direct modulation of stable microtubules by Spastin. Any subtle changes in stable microtubules by spastin might affect potential trafficking machinery which is responsible for the apical Crb targeting. But, these two possibilities are not necessarily mutually exclusive.

Spastin has microtubule-severing activities *in vitro*. Therefore, microtubule-severing activity of Spastin may facilitate the apical Crb domain, since loss of spastin caused loss of Crb, and gain of spastin caused the expansion of the Crb domain. Furthermore, this facilitating activity of Spastin for the apical domain could be independent from the main stable microtubule structures which are located far beneath the apical domains (Figure 2E). This possibility is supported by the more direct influence on apical Crb, as the stable microtubules were not dramatically changed, relatively speaking, by either spastin mutants or spastin overexpression (Figures 4 and 5).

During the massive growth of the rhabdomeres in the pupal retina, many membrane materials, including Crb, will be targeted into the growing apical membranes. Spastin may participate in this material transport to the apical membrane domain during rhabdomere growth, although the initial targeting is spastin-independent. The outcome of this study will provide useful information for understanding the molecular genetic mechanism of spastic paraplegia. Although the spastin mutation subtly affects the main microtubules, this genetic approach will provide more convincing clues concerning the microtubule-based processes in photoreceptor morphogenesis.

Thus, analysis of the microtubule-modulating Spastin in *Drosophila* photoreceptors may provide important insights into the understanding of the functional basis of the microtubule-based structure and the microtubule-related genes involved the formation and development of photoreceptors. Evolutionary conservation in the structure and function of eye morphogenesis genes makes the *Drosophila* eye an excellent model for studying the genetic and molecular basis of retinal cell organization.

Future work will help to uncover other genes that might affect the microtubule cytoskeleton and cell polarity targeting during the extensive membrane growth phase of the pupal eye. Determining the role of Spastin in photoreceptor development will help in understanding retinitis pigmentosa, spastic paraplegia and other retinal degenerative diseases that involve mutations in *crb* and *spastin*. The finding that human mutations in CRB1 lead to retinitis pigmentosa [15] emphasizes the importance of deciphering the molecular networks associated with Crb in the apical membrane domain of the *Drosophila* photoreceptor.

In summary, we examined the role of Spastin in the regulation of the apical Crb domain in developing photoreceptors. Our data strongly suggests that Spastin plays important functions in the modulation of cell membrane domains including the apical domains of photoreceptors during pupal eye development. Because proper maintenance of the apical Crb domain is important for the massive growth observed in rhabdomeres at the apical region of photoreceptor cells, malfunction of spastin results in severe defects in the formation of functional photoreceptors.

## Methods

### Genetics

Mitotic recombination was induced by using the FLP/FRT method for clonal analysis [25]. s*pastin^5.75^* mutant [10] clones were produced by eye-specific expression of FLP in *y w ey-Flp/+; spastin^5.75^ FRT82B/FRT82B Ubi-GFP*. Overexpression of *spastin* was induced by crossing *UAS-spastin* [10] with *GMR-GAL4* [28].

### Immunohistochemistry

The following primary antibodies were used: mouse anti-Acetylated tubulin (Sigma), 1∶1000; mouse anti-α-tubulin (Sigma), 1∶500; mouse anti-β-tubulin (Sigma), 1∶500; rat anti-DE-cadherin (DSHB), 1∶10; rat anti-Crb [15], 1∶400; rabbit anti-Dlg [29], 1∶1,000; rat anti-Dpatj, 1∶500 [30]; sheep anti-GFP (Biogenesis), 1∶100; and guinea pigs anti-Spastin [10], 1∶300; rabbit anti-Sdt, 1∶500 [31]. Secondary antibodies conjugated with Cy3, Cy5, or FITC were from Jackson Laboratories. Fluorescent immunostaining and confocal analysis of pupal eyes were carried out as reported [13], [26], [32], [33], [34]. Specificity of the antibody staining of Spastin was verified by clonal analysis using the protein-null mutant [10]. Fluorescent images were acquired on an Olympus FV1000 confocal microscope. Image analysis and quantification were performed using ImageJ and Adobe Photoshop.
